# Radiation risks knowledge in resident and fellow in paediatrics: a questionnaire survey

**DOI:** 10.1186/s13052-015-0130-x

**Published:** 2015-03-22

**Authors:** Sergio Salerno, Paola Marchese, Andrea Magistrelli, Paolo Tomà, Domenica Matranga, Massimo Midiri, Alberto G Ugazio, Giovanni Corsello

**Affiliations:** Section of Radiological Sciences, DIBIMEF, University Hospital Policlinico, University of Palermo, Via del Vespro 127, 90127 Palermo, Italy; Department of Diagnostic Imaging, Children’s Hospital Bambino Gesù, Rome, Italy; Department of Sciences and for the Promotion of Maternal and Child Health G. D’Alessandro University Hospital Policlinico, University of Palermo, Palermo, Italy; Department of Pediatric Medicine, Children’s Hospital Bambino Gesù, Rome, Italy

**Keywords:** Radiation protection, Paediatric residents, Paediatric fellows, Education and training

## Abstract

**Background:**

Analyse through a multi-choice anonymous questionnaire the knowledge’s level in paediatric residents and fellows in two different main Italian hospital, looking mainly to the information to patients and relatives related to risks of ionizing radiation used in common radiological investigations in children.

**Methods:**

65 multi choice questionnaires were distributed to paediatric residents and fellows of two different hospitals, an University Hospital (A.O.U.P. “P. Giaccone”- University of Palermo) and a national reference centre for paediatrics (Ospedale Pediatrico Bambino Gesù - Rome). The questionnaire included twelve multiple-choice questions with the aim of analyzing the knowledge about ionizing radiation related risks in infants and children who undergo common diagnostic radiology investigations. The data obtained were processed using software Stata/MP version 11.2. In order to measure the level of expertise of each interviewee a binary indicator was built. The value 1 was assigned if the percentage of correct answers exceeds the median of the distribution and 0 for values not exceeding the median. The association between the level of competence and demographic characteristics (gender, age) and training experience was measured by means of α^2^ test.

**Results:**

51/65 questionnaires were completed, returned and analysed (87.7%). Only 18 surveyed (35%), (95% IC = [22%-48%]) can be defined as competent in radiation risk knowledge for common radiological investigations, considering the percentage of correct answers at least of 50% (sufficient knowledge was given with a minimum score of 8 correct answers out of 12).

**Conclusions:**

The study demonstrates an urgent need to implement the radiation protection knowledge in the training programme of paediatricians, that improve if just a short targeted training is performed.

## Background

Ionizing radiation techniques are fundamental in medical practice, particularly in paediatrics. The use of imaging, especially of multidetector computerized tomography (MDCT) has grown up considerably in recent years, especially in emergency care [1-5]. According to the Directive 2013/59/EURATOM OF THE COUNCIL of December 5, 2013 [6], each medical exposure to radiation must be justified and patients have to be properly informed.

This Directive put great emphasis on exposure of the paediatric population, just because of their remarkable radiosensitivity [7-9]. It is very important for paediatricians to know general issue on radiation protection. These data unfortunately are not available and/or not well known, as well as dose reference levels (DLR) for paediatric population (that are not available in the majority of the European countries, as well as in Italy, according to dosedatamed2 project) [10-12].

Otherwise the number of paediatric diagnostic procedures is well known and constantly growing [2]. According to CT Benchmark Report 2007 in United States (US) on a total of 68 million MDCT examinations, 11% were performed on a paediatric population [3]. In Europe the frequency of paediatric MDCT exams is ≈ 2% [10], in particular in Italy this percentage is around 2,2% [13].

The increasing number of diagnostic examinations that use ionizing radiation on paediatric patients, along with some alarming reports on use of radiation on newspaper and web, lead to the need for a proper information to patients and relatives [14,15]. Indeed, before each diagnostic exam implying ionizing radiation, the patient and relatives need to be well informed on dose, potential health risks and risk benefit ratio by referring physician and radiologist [16]. So deep knowledge on radiation risks is mandatory in referring physicians, especially for paediatricians [5,16].

Despite this evidence many articles have shown a not sufficient knowledge on radioprotection in general practitioners and different specialists in several countries [17]. Also the level of awareness among paediatricians on procedure that requires ionizing radiation in children, is not yet well known in Italy.

The aim of the present paper is to assess, on paediatrics residents and fellows, the level of knowledge on possible radiation risks and its balance and skills to inform parents and relatives of the children in case of prescription/requirement of common diagnostic imaging exams, using a multi choice questionnaire.

## Methods

**Figure 1:**
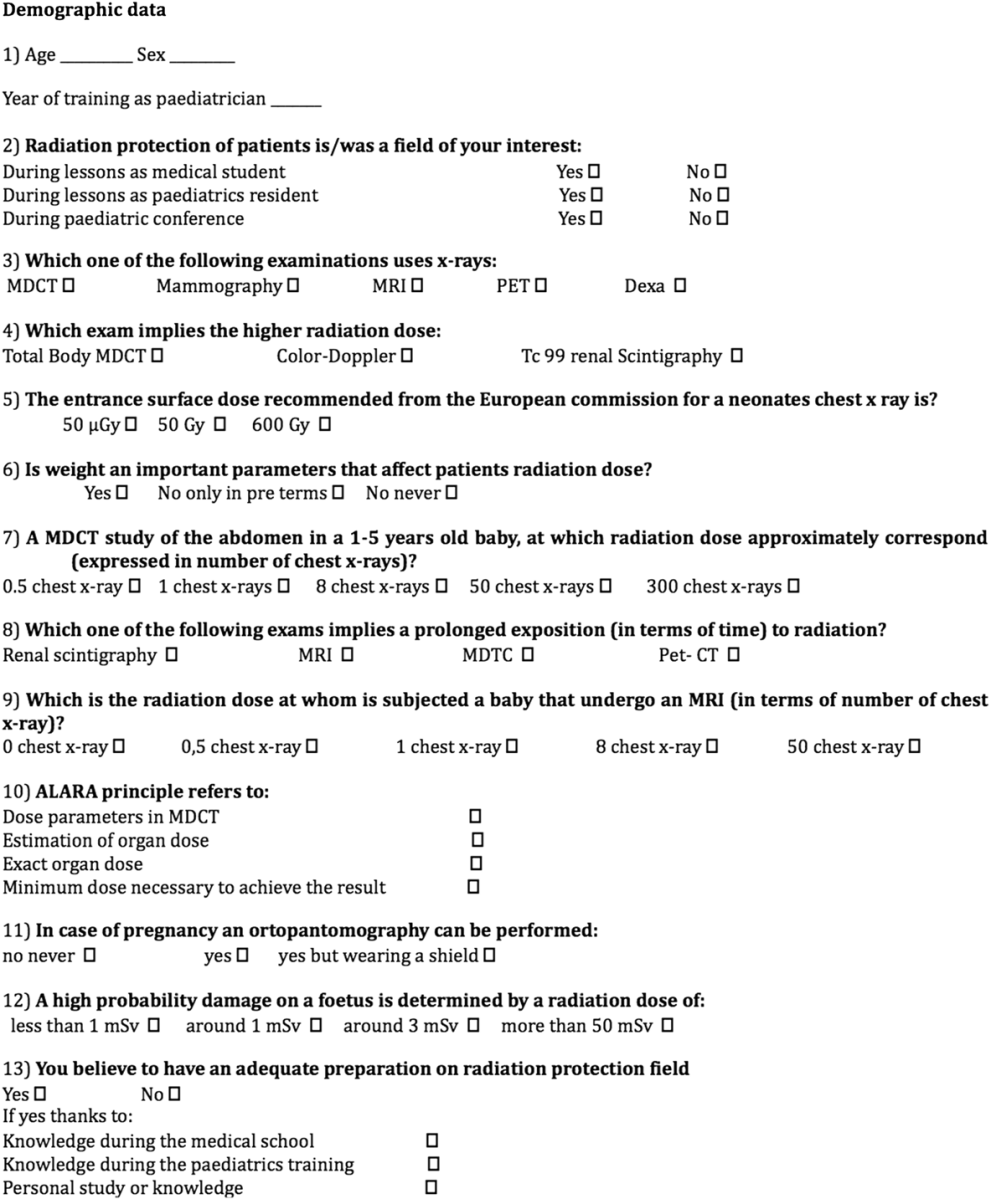
**Questionnaire on radiation protection and dose knowledge in common paediatric diagnostic examinations.**

We conducted a prospective study proposing a multiple-choice questionnaire (Figure 1), to 65 paediatrics residents and fellows of two different centres, a University Hospital (A.O.U.P. “P. Giaccone”- University of Palermo) and a national reference centre for paediatrics (Ospedale Pediatrico Bambino Gesù - Rome). The questionnaire was designed, using previous experience reported in the literature [17,18], to determine the participant’s knowledge about general concepts of radiation protection and risks related to radiation exposure, in common radiologic investigations in paediatric/childhood.

The questionnaire consisted in 12 questions and was divided into two sections: the first part required to be filled with personal demographic data and the second part consisted of specific questions about radiation protection (Figure 1).

In particular, the first three questions were composed of demographic data such as age, gender (values 0 and 1 respectively assigned to female and male), professional status (value 0 for resident on 1^st^ year, value 1 for resident of higher years, value 2 for fellow). The following questions investigated knowledge on common measure of radiation protection and doses in common radiological investigations, including MDCT scans and nuclear medicine. Last question was a self-assessment about personal knowledge about “the risk dose in radiology”. The questionnaires were handed out during multidisciplinary team meeting, by radiologist resident that request the participants to fill it anonymously and individually and return it back in the same day.

**Figure 2:**
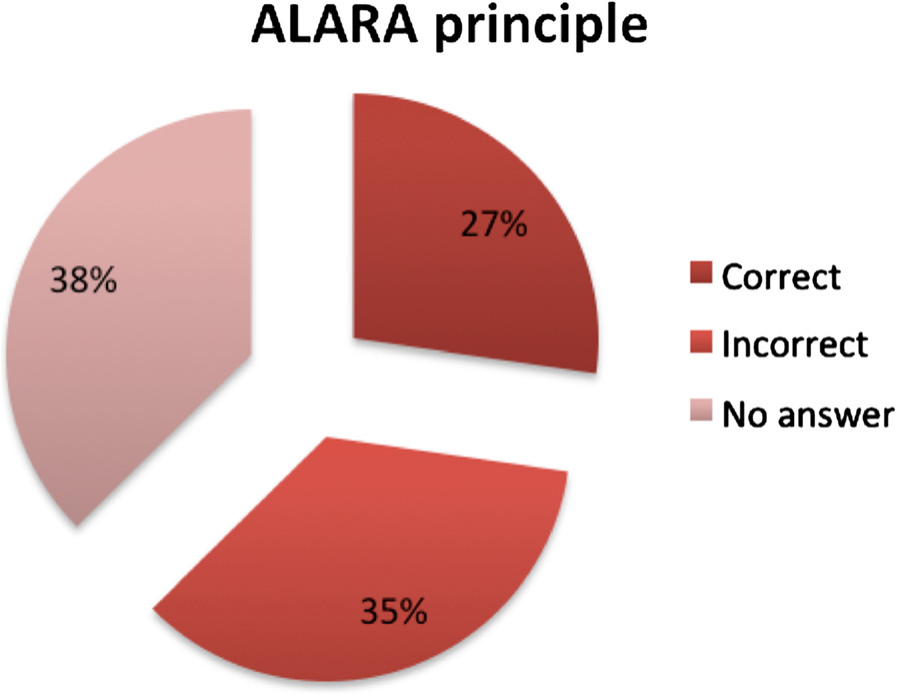
**Percentage of correct answers on the question on ALARA principle.**

**Figure 3:**
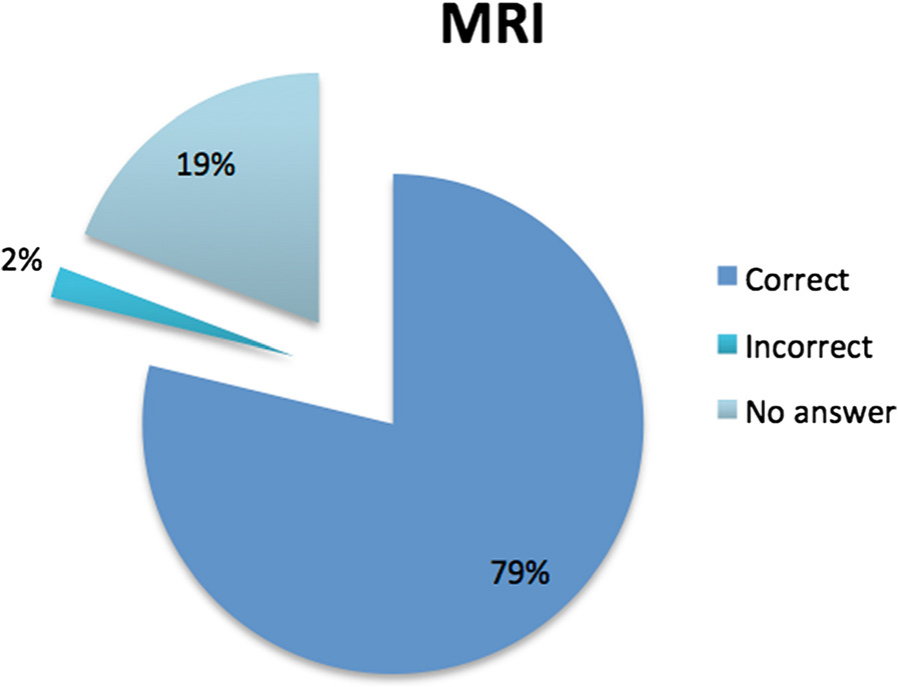
**Answers to the question about the MRI involves the use of X-radiation.**

**Figure 4:**
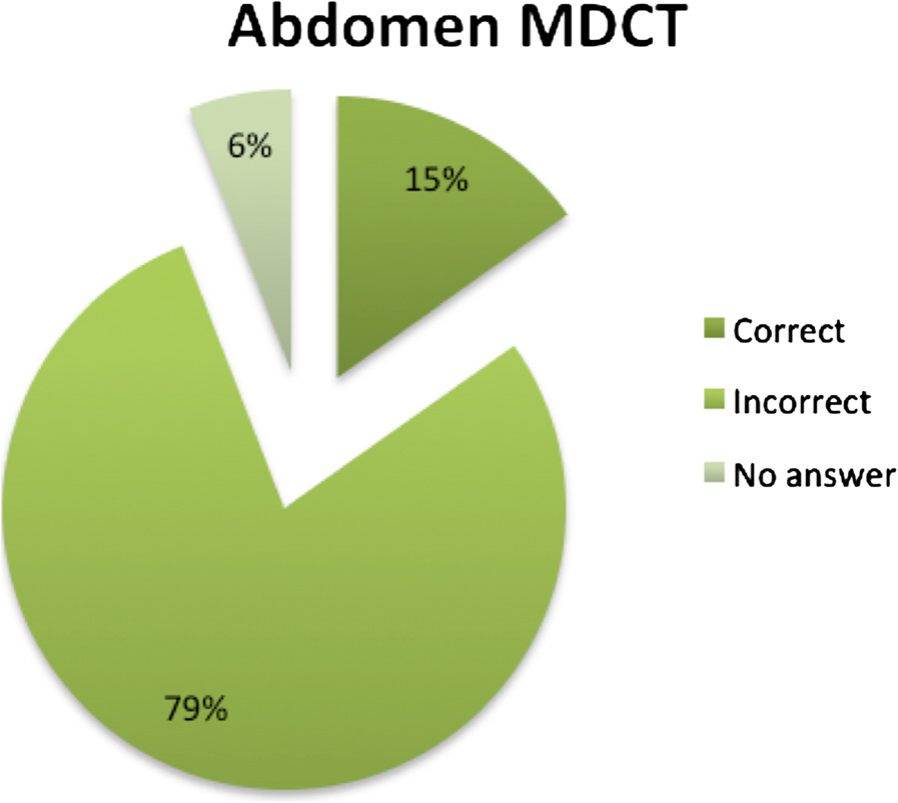
**Percentage of different types of answers on the question about the possible radiation dose amount of abdomen MDCT scan in a child (1–5 years) (expressed in number of chest x-rays).**

**Figure 5:**
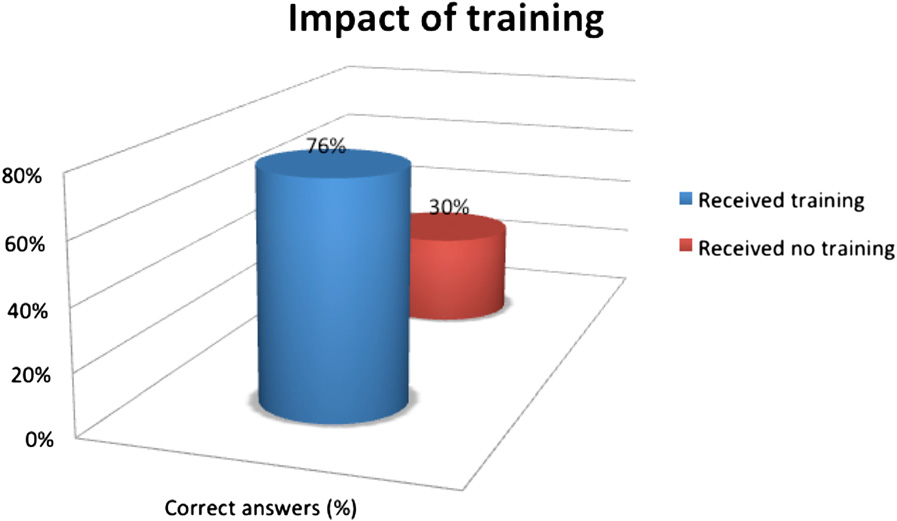
**Comparison with trained participants and not fully trained participants.**

Data after analysis of the filled questionnaires were reported as graphs, percentages and averages. (Figures 2, 3, 4 and 5). The numerical value of 1 was assigned to each correct answer, the value 0 to each wrong answer, the value 2 was given to each question with no response.

To measure the level of expertise of each participant in the survey, a binary indicator was built; with the value of 1 if the percentage of correct answers exceeds the median of the distribution and 0 for values not exceeding. The median value was set on value of correct answers equal to 8. All participants with number of correct answers under 8 have been considered insufficiently competent, on the other hand participants who reached at least a number of correct answers equal to 8 were considered competent.

The association between the level of competence and demographic characteristics (gender, age), position (resident, fellow) and training was measured by means of *X*^2^ test. The statistical analysis was performed with the statistical software Stata/MP 11.2.

## Results

On a total of 65 residents and fellows available in the two centres involved, only 51 completely filled the questionnaire (87.7%). The residents and fellows population was constituted by: 6 trainees of the first year; 26 trainees of the second and third year and 19 fellows, who have recently completed the training (Table 1). The mean age of the participants was 36 years old.

**Table 1:** **Sample characteristic paediatrics residents and fellows**

	^**(1)**^ **N correct answer**	^**(2)**^ **Minimum of 50% of correct answer (%)**	***Prob.***
**Age**			0.045
≤28	14 (8)	57%	
>28	37 (10)	27%	
**Year of training**			0.223
1st year of paediatrics residency	6 (4)	66%	
Higher year of paediatrics residency	26 (9)	33%	
Fellow	19 (5)	26%	
**Lessons focussed on radiation protection during the medical school** ^**(1)**^			0.003
Not	13 (4)	30%	
Yes	38 29)	76%	
**Lessons focussed on radiation protection during the residency training**			0.867
Not	39 (27)	69%	
Yes	12 (8)	67%	
**Conference focussed on radiation protection**			0.915
Not	45 (16)	35%	
Yes	6 (2)	33%	

^**(1)**^ **Between () the number of correct answer in the sample.**

^**(2)**^ **Percentage of correct answer compared to the demographic data and year of training.**

74% had regularly attended radiation protection lessons (as part of the Radiology course) during the medical school/study. 23% of trainees followed also conference focussed on radiation protection during residency course (Table 1).

No significant differences were found in the percentage of correct answers in relation to sex and years of training, but the percentage of correct responses in younger subjects was significantly higher (p = 0.045). A significant correlation was found in participants that followed conference on radiation protection, indeed they reported a higher percentage of correct answers (p =0.025) (Table 1). According to the definite cut off value, 35% of the participants were considered competent (n = 18) (95% IC = [22%-48%]) (Table 1). In details, 21% of participants were not aware that MRI does not use ionizing radiation (Figure 3); 78% (95% IC = [66%-89%]) were not able to define an average of radiation dose (expressed in numbers of chest x-rays) in a child (1–5 years) submitted to a MDCT scan of the abdomen; 78% were not able to answer to the ionizing radiation technique that implies a more prolonged exposure, in terms of time (Table 2).

**Table 2:** **Distribution of the correct answer for single questions in the questionnaire completely filled**

**Questions**	**Answers n (%)**		
	**Correct**	**Incorrect**	**Do not know/no answer**
MDCT examination involves the use of X-rays?	50 (98%)	-	1 (1,9%)
Mammography involves the use of ionizing radiation?	44 (86%)	4 (7,8%)	3 (5,8%)
MRI involves the use of ionizing radiation?	40 (78%)	1 (1,9%)	10 (19%)
PET involves the use of X-rays?	14 (27%)	28 (54%)	9 (17%)
DEXA involves the use of X-rays?	18 (35%)	21 (41%)	12 (23%)
Which exam involves higher exposure to ionizing radiation among: total body MDCT, Color Doppler, renal scintigraphy?	42 (82%)	9 (17%)	-
What is the dose (Entrance surface dose) recommended by the European Commission in the diagnostic reference levels for a chest x-ray in a new born?	21 (41%)	19 (37%)	11 (21%)
IS the weight an important parameter that affect patient radiation dose?	29 (56%)	20 (39%)	2 (3,9%)
A MDCT scan of the abdomen in a child (1–5 years) correspond to a radiation dose of (expressed in number of chest x-ray)	8 (15%)	40 (78%)	3 (5,8%)
Renal scintigraphy involves prolonged exposure to ionizing radiation (in terms of time)?	8 (15%)	40 (78%)	3 (5,8%)
Radiation dose of a child submitted to MRI is (expressed in number of chest x-ray)	43 (84%)	7 (13%)	1 (1,9%)
ALARA principle refers to	14 (27%)	18 (35%)	19 (37%)
During pregnancy an ortopantomography can be easily performed?	20 (39%)	19 (37%)	12 (23%)
Foetus radiation damage is determined with high probability for a dose of?	13 (2,5%)	29 (56%)	9 (17%)

Other important data regards lack of knowledge in general principle of radiation protection as: possible risks of radiation exposure (orthopantomography) during pregnancy (37%) (95% IC = [26%-52%]) (Table 2); radiation dose associated with a high probability risk of foetal malformation (56%) (95% IC = [42%-70%]), (Table 2).

## Discussion

The awareness of the issues of radiation protection among different clinicians is generally low, with widespread underestimation of doses and risks [17]. As in other published experience, knowledge in radiation protection is poor in comparison to the increased need of information by patients and/or relatives [18]. This lack of awareness of ionizing radiation dose exposure is particularly important in relation to the high number of patients who receive inappropriate or repeated diagnostic examinations.

The increasing use of MDCT in the world, particularly in younger population, is becoming a serious problem in radiation protection and awareness of the risks [19-22]. On the basis of empirical data a proper balance is crucial for the safety assessment compared to the benefits that MDCT provides [23,24]. It is imperative for physicians to have an idea of radiation dose involved in common imaging investigations and possible risks to radiation exposure, to be able to properly inform patients and relatives [19]. Over the past 15 months, three large epidemiological studies have assessed the risk of cancer due to MDCT scans in children [20-22]. Miglioretti et al. [20] investigated the rates of MDCT scan use in a large population of children, estimating effective dose in children and calculating excess lifetime cancer risk attributed to MDCT scans. Other two studies [21,22] provided data on the number of MDCT scans in large paediatric populations and retrospectively analysed possible excess lifetime cancer risk attributed to CT scans.

Despite the immediate benefit (major diagnostic accuracy and scanning speed of MDCT compared with traditional radiography) for the individual patient is significantly high overcoming the long-term risks related to the radiation dose, the higher dose given with MDCT compared with traditional radiography, have raised several concerns about children’s health [23-25]. Especially considering the annual increase rate of MDCT examinations in paediatric population; in particular the significant increased use in emergency, recording more than 40% every 5 years in some countries [3,24].

The first serious measure to be implemented to limit the radiation-induced risk is the reduction of the prescription of diagnostic examinations that have low or no utility in the diagnosis and/or detection of disease in paediatric patients by general practitioners and/or paediatricians [25]. To date, the problem has not yet been investigated in Italy nor between the possible prescribers (general practitioners, specialists, dentists), neither among subjects belonging to radiological area, although since 1995 Italy has built up good legislation intended to radiation protection of the patient, with required courses for medical staff [26].

Puri et al. [27] showed that a large proportion of emergency doctors are unaware of the risk associated with commonly performed MDCT scans, but doctors with broader experience, despite the shortcomings in the knowledge of risk associated with radiation, are also more likely to consider the radiation dose of patients, to conduct an analysis of the risk-benefit and are less likely to require a MDCT scan if it is not necessary. This study revealed that the clinical experience is significantly associated with a beneficial behaviour towards the use of MDCT, more that knowledge of the risk of cancer attributable to radiation.

A letter to the editor in the British Medical Journal [28] firstly has emphasized the inadequate training in radiation protection of medical staff in a large health district in the UK, despite the UK have introduced from many years a serious medical education program continuous (CME) and published a manual of radiology having as one of the main objectives the reduction of the dose to patient [29]. An unsatisfactory low knowledge was also reported in a group of specialist radiologists and radiographers by Foley [30], with a questionnaire regarding the MDCT parameters and their influence on patient dose and image quality; the author reported a correct response rate of 22% in the group of radiologists and 32% in the group of technicians. Also knowledge among cardiologists showed to be suboptimal, but can greatly improve with a focused effort to training and teaching [31]. In addition the guidelines of the European Community in the field (EC - Medical Exposure Directive), recommend to the member states to introduce courses on radiation protection in the basic training curriculum of surgeon and dentist, and recently in all physician courses [6].

For the reasons stated above and for the widespread ignorance on the subject highlighted by many authors [32-35] we decided to carry out this survey among the paediatric residents and fellows. The result was in line with that reported in the literature, and therefore not very comforting, as well as evidenced among the junior/young doctor in Ireland, where the 8% of respondents thinks that ultrasounds are ionizing radiation [33] and the 14% of the German Ruhr paediatricians who thinks that the Magnetic Resonance uses ionizing radiation [34]. The knowledge gap concerning radiation doses and associated health risks among physicians is evident in different publications according to a systematic review conducted by Krille [35], but some of the answers of his questionnaire induce some considerations on actual radiation protection knowledge.

Major concern also raises the lack of knowledge of dose level during pregnancy potentially harmful to the foetus, and the possibility or not to obtain an orthopantomography in pregnant woman. Even in USA, where MDCT were widely used in paediatric population, for example in the diagnosis of appendicitis, are rediscovering a strong impulse to radioprotection culture. This has resulted in an increase in image wisely campaign [36]. For Walshe [32] the “knowledge gap” is an international problem and therefore requires an international response. The American College of Radiology launched different campaigns for appropriate imaging [37,38], to increase awareness of the risk of ionizing radiation and try to reduce unnecessary imaging, with particular reference to paediatric imaging. We found a interesting evidence: the frequency of lecture courses and/or conferences of radiation protection by students and/or trainees clears the gap. In fact, the percentage of correct answers to at least half of the questions rises to 76% if students have attended at least one radioprotection lesson during the residency and 67% if they followed lessons focussed on radiation protection in the medical school. Thus demonstrates that proper education can drastically reduce radiation protection knowledge gap. So if in one side there is a growing awareness among paediatric radiologists about potential risks associated with ionizing radiation in medical imaging, on the other this study suggests that there is, among paediatricians, still widespread underestimation of doses and risks. Some corrective measures should be implemented such as: make radiation protection lessons “more attractive” in radiology course during medical school, probably “be more practise and less radio biological”. Stimulate the radiation protection issue, because/since only administration of the questionnaire causes an increased demand of information. An important element of radiation protection is to guarantee that physicians have sufficient knowledge to enable them a balanced and accurate assessment of the risk-benefit ratio when considering radiological examinations. Paediatric radiologists mission have to be to inform and educate other colleagues about radiation protection and give literature contributions on the field to play an important role in raising awareness [39].

## Conclusions

The physicians surveyed of our study were from two different hospitals in two different regions. Therefore our results may not be applicable at national level; nevertheless it demonstrated that paediatricians with different school source have an insufficient knowledge on risks implied on commonly radiological investigations.

There is a need for better training in radiation protection and its could be obtained with a joint programme involving the Italian Society of Radiology and the Italian Society of Paediatrics and Neonatology to increase knowledge among physicians.
